# Breastfeeding practices 2008–2009 among Chinese mothers living in Ireland: a mixed methods study

**DOI:** 10.1186/s12884-019-2713-9

**Published:** 2020-01-23

**Authors:** Qianling Zhou, Katherine M. Younger, Tanya M. Cassidy, Wenyi Wang, John M. Kearney

**Affiliations:** 1 Department of Maternal and Child Health, School of Public Health, Peking University, Beijing, China; 2 School of Biological Sciences, Dublin Institute of Technology, Kevin Street, Dublin 8, Republic of Ireland; 3 School of Nursing, Psychotherapy and Community Health, Dublin City University, Dublin, Republic of Ireland; 4 Johns Hopkins University, Baltimore, USA

**Keywords:** Breastfeeding, Infant feeding, Chinese, Ireland, Migration, Mixed methods research

## Abstract

**Background:**

Migration to another country has a potential influence on breastfeeding practices. A significant difference in breastfeeding rates between Irish nationals and non-nationals has been reported. This study was conducted to explore breastfeeding practices of the Chinese in Ireland, one of the largest Irish ethnic groups, and to explore the influence of living in Ireland on breastfeeding practices. This is the first and the only migration study so far on breastfeeding practices among the Chinese in Ireland.

**Methods:**

A sequential explanatory mixed methods approach was adopted. The first phase was a cross-sectional self-administered retrospective mailed survey, to explore breastfeeding practices and determinants of breastfeeding among a convenience sample of Chinese mothers living in Ireland (*n* = 322). Recruitment was conducted in the Dublin metropolitan area, with the application of the snowball technique to increase sample size. The second phase consisted of seven semi-structured focus groups (*n* = 33) conducted in Dublin, to explore the influence of living in Ireland on breastfeeding among Chinese mothers who had given birth in Ireland. Quantitative data were analyzed by univariate and multivariate logistic regression analyses, and informed the qualitative data collection. Qualitative data were analyzed by thematic content analyses, to explain and enrich the qualitative results.

**Results:**

The breastfeeding initiation rate among Chinese immigrants to Ireland who gave birth in Ireland (CMI) (75.6%) was high and close to that of Chinese immigrant mothers who gave birth in China (CMC) (87.2%). However, giving birth in Ireland was independently associated with a shorter duration of breastfeeding (< 4 months) among Chinese immigrants. Qualitative results explained that a shorter breastfeeding duration among CMI than that of CMC was mainly due to cultural conflicts, a lack of family support, language barriers, immigrants’ low socioeconomic status, and mothers’ preference for infant formula on the Irish market. Both quantitative and qualitative data revealed a strong cultural belief in the efficacy of the traditional Chinese postpartum diet for breast milk production for both CMC and CMI. Antenatal feeding intention was a strong determinant for breastfeeding initiation and duration among CMI.

**Conclusion:**

Migration to Ireland was found to be associated with a shorter duration of breastfeeding of the Chinese. Culturally sensitive and language-specific education and support of breastfeeding is needed for the Chinese mothers living in Ireland. The mixed methods design presented here might serve as a template for future migration research on breastfeeding.

## Background

Breastfeeding is the optimal feeding practices for infants as it provides unique combination of nutritional, anti-infective, immunological and psycho-physiological benefits. The protective effects of breastfeeding against infectious diseases in infancy [1], childhood obesity [2], and chronic cardiovascular and metabolic diseases in adulthood [3] have been demonstrated. The World Health Organization (WHO) has recommended exclusive breastfeeding for the first 6 months of life. Thereafter infants should receive complementary foods with continued breastfeeding up to 2 years of age or beyond [4]. The WHO has targeted to increase the rate of exclusive breastfeeding in the first 6 months up to at least 50% by the year 2025 [5], and called upon all facilities providing maternity and newborn services worldwide to implement the ‘Ten Steps to Successful Breastfeeding’ (a package summarizing policies and procedures to support breastfeeding that all baby friendly hospitals are required to follow) [6, 7].

Breastfeeding is influenced by multiple factors, including maternal demographic, social, cultural, biomedical and psychological factors [1, 8, 9]. Migration to another country may induce changes in breastfeeding practices because the social environment in the host country might be less breastfeeding friendly (e.g. less public facilities for breastfeeding, breastfeeding is not the cultural norm in host country) in comparison to that in their home country. Lower prevalence of breastfeeding has been found in Asian immigrants living in Western countries, in comparison to their counterparts in home countries [10–14]. While the majority of mothers in China choose to breastfeed [15], studies in the past three to four decades report that immigrant Chinese mothers in Europe or North America seldom breastfeed their children [16, 17]. Even though breastfeeding initiation rates remain high for Chinese mothers living in Australia; early cessation of breastfeeding has been revealed [18]. The perceptions of inadequate breast milk [12, 18] and inconvenience of breastfeeding are prevalent among the Chinese immigrants [19, 20]. No information related to breastfeeding is available in the recent decade among immigrant Chinese in Europe.

The Chinese postpartum tradition of ‘*doing the month*’ has a potential positive influence on breastfeeding practices in the contemporary society [21]. The first 1 month after childbirth is considered as a critical and vulnerable period for the newborn and the mother. A number of behaviours restrictions (e.g. avoid drinking cold water) should be followed and a special diet should be consumed by the mothers [22]. The consumption of a special postpartum diet has been traditionally regarded as beneficial to breast milk quantity and quality. Such cultural belief is still prevalent among Chinese mothers living in China and abroad in recent decades [21, 23]. Migration to another country might result in maternal difficulties in the consumption of postpartum diet, which might have a negative impact on breastfeeding.

Ireland’s breastfeeding initiation rate (44–56%) is among the lowest in the world [3, 24–26]; despite of some gradual increases over the last 10 years [27]. The Growing Up in Ireland national cohort reveals that about 56% of the children born in Ireland have ever been breastfed [28]. Exclusive breastfeeding rates at hospital discharge (46.3%) [25] and at 6 months postnatal (15%) [29] in Ireland are also very low. A 5 year breastfeeding action plan (2016–2021) has recently been published to improve breastfeeding rates and support mothers to breastfeed in Ireland [7].

The population of non-nationals in Ireland has increased by 87% from the year 2002 (224,261 persons) to 2006 (419,733 persons). The number then stabilized at 544,357 persons in 2011 [30]; but the most recent census data suggests these figures are rising again [31]. A national infant feeding survey has revealed significant differences in breastfeeding initiation rates between Irish nationals (50%) and non-nationals (76%). The study also stressed a need to understand the breastfeeding practices of non-Irish women in Ireland [32]. Such information is useful to identify target groups and develop appropriate strategies for breastfeeding interventions, which are important for the overall improvement of breastfeeding practices in Ireland.

The Chinese, who comprise 3% of the non-nationals, represent one of the largest ethnic groups in Ireland [33]. The Chinese immigrants have contributed to about 17% of the increase of the non-Irish population from 2011 to 2016 [31]. The majority of the Chinese immigrants are living in Dublin City and its suburbs (67%), and are college students (43%) or employees (44%) [33]. More than 85% of Chinese women living in Ireland are of child-bearing age (20–39 years old) [33]; however, there is no published literature on breastfeeding practices among this population sub-group.

This present study was undertaken to explore the breastfeeding practices of the Chinese immigrants in Ireland, to identify the determinants of breastfeeding initiation and duration, and to gain an in-depth understanding of the influence of migration to Ireland on breastfeeding practices among Chinese mothers who had given birth in Ireland.

## Methods

### Definition

In this paper, breastfeeding is indicated as ‘any breastfeeding’, and defined as feeding the child with any breast milk [34]. Breastfeeding initiation in this paper is defined as whether the child was put to breast at least once after birth or was ever fed with breast milk.

### Study design

A mixed methods research design was adopted in this study. A combination of qualitative and quantitative research elements was used to enhance the breadth and depth of understanding of this research topic [35]. The rationale for the application of mixed method approaches is that factors influencing breastfeeding practices are multifaceted; and such design is appropriate to study the complexity of many different factors that influence health and illnesses [36].

This was a two phases, sequential explanatory mixed methods study, in which the quantitative data informed the qualitative data collection; and the qualitative data explained, supplemented and enriched the quantitative results [37]. Phase 1 (i.e. the quantitative phase) was a cross-sectional survey, conducted to explore the prevalence and determinants of breastfeeding practices among Chinese mothers in Ireland. Results from Phase 1 suggested a need to further understand factors influencing breastfeeding duration. Phase 2 (i.e. the qualitative phase) was focus group interview, among Chinese mothers who have given birth in Ireland. Content analysis was performed to explain and elaborate on the quantitative findings. Both quantitative and qualitative results were finally integrated when drawing the conclusions [38].

This study was approved by the Research Ethics Committee of the Dublin Institute of Technology.

### The participants

Inclusion criteria for the participants in both Phase 1 and Phase 2 were Chinese women who were born in China, had given birth to at least one child, and had been living in Ireland for at least 6 months at the time when they were surveyed and/or interviewed. For Phase 2, participants additionally had to have given birth in Ireland.

### Setting and data collection

#### Phase 1

Phase 1 was conducted between September 2008 and March 2009. As the majority of Chinese immigrants were residing in Dublin city and its suburbs [33], fieldwork was conducted in the Dublin. A convenience sampling strategy was adopted in recruitment. The study was advertised in three venues: a) a Chinese newspaper distributed in all Irish counties, and websites well-known to the Chinese in Ireland; b) a study poster put up in two large Chinese supermarkets in Dublin city centre; and c) announcement sent by the Chinese community leaders to their members via regular community events or individual contacts.

Concurrent with advertisements stated above, potential participants were approached by the researcher (QZ) in person at places frequented by Chinese mothers in Dublin and surrounding suburban areas (including Chinese supermarkets, Chinese language schools, a Chinese cultural music and dancing school, church organizations and Chinese restaurants). In the Chinese supermarkets and restaurants, the researcher approached to women who walked through; while in the schools and church organizations, teachers and the church activity organizers gathered the mothers, helped announce the study and encouraged mothers to participate in the study before the researcher approached to them individually. Permission to approach mothers was sought from the Chinese supermarket, restaurants, schools and church organizations. Eligibility of the participants was briefly assessed by the researcher at recruitment. The eligible ones were explained the purpose of the study and assured of total anonymity and confidentiality, with the provision of a Chinese information sheet. They were given a few minutes to read the information sheet and consider whether or not to participate in the study. If they agreed to participate, the participants were given a questionnaire together with a stamped addressed envelope, and were asked to complete and return the questionnaire by mail. The returned questionnaires were checked for consistency and completion, and double checked for eligibility of the participants. A follow-up telephone call was made to participants who failed to complete the questionnaire or who provided unclear information. A ‘snowball’ technique was used to increase the sample size, i.e. participants were invited to help announce and distribute the questionnaire (together with the consent form) to their friends and family members who met the inclusion criteria, regardless of whether they were living in Dublin or other Irish counties. No particular efforts were made to control regional or urban-rural variations of the sample. A 5-euro shopping voucher was posted to each participant upon the completion of the study as an incentive. Written informed consent was obtained from all the participants before any assessments.

#### Phase 2

Phase 2 was conducted between September and October 2009 in Dublin. The first author QZ, who was a female doctoral student (in public health) in Ireland conducted the focus groups. She speaks Chinese as her mother language, and had received intensive training in qualitative research. The author TMC, an anthropologist with rich qualitative research experience in lactation, guided the development, implementation and analyses of this qualitative study. A semi-structured interview guide was developed, to elicit the barriers to the initiation and continuation of breastfeeding in Ireland. The guide started with an open question on mothers’ personal experience in childbirth and infant feeding in Ireland. Mothers were prompted to share how they initiated and sustained breastfeeding, and why they terminated breastfeeding. Mothers who had previously given birth in China to an older child were additionally asked to compare their breastfeeding experience in China versus in Ireland. The guide was developed based on the breastfeeding literature, some findings of Phase 1 (breastfeeding duration of CMI was significantly shorter than that of CMC, determinants of breastfeeding initiation and duration of CMI), and suggestion of the research team members, including three breastfeeding researchers, an anthropologist, and a Chinese medical doctor. Two Chinese mothers in Ireland who were not the study participants read the interview guide and confirmed the literacy of the questions.

Participants consisted of mothers recruited from Chinese language schools (*n* = 14) and mothers who were the participants of Phase 1 (*n* = 19). On one hand, the researcher approached to mothers who were waiting for their children during the language classes in person, and invited the eligible ones to participate in Phase 2. Permission to approach mothers was sought from the Chinese language schools. On the other hand, mothers of Phase 1 who met the inclusion criteria of Phase 2 were randomly selected and contacted by telephone call. The majority of mothers approached had been aware of the research and some had developed a rapport with the researcher. All mothers invited (*n* = 33) supported the research and agreed to participate in Phase 2 if the time and study location suited. Eligibility of the participants was assessed by the researcher at recruitment. Participants from the same region of hometown were purposively allocated into the same focus group, as they might have similar cultural belief and health practices, which allowed them to understand each other well. Each focus group had four to six participants. All groups were taped-recorded and conducted in Chinese and in a location convenient to participants (e.g. quiet classrooms of language schools). Nobody else presented in the interview besides the participants and the researcher. Groups commenced with an introduction to the research and instructions of the discussion. Confidentiality was assured. At the end of the focus groups, participants were required to complete a brief questionnaire that sought demographic information. Field notes were taken immediately by the facilitator (QZ) to highlight areas of key significance in the discussion. Audiotapes were transcribed verbatim in Chinese. Qualitative data (in Chinese) were generated from both transcriptions and field notes. Data collected was considered as saturated because redundant information appeared and no new information was discovered in the last focus group. Written informed consent was obtained from participants before the interview.

### Measures

#### Phase 1

In Phase 1, a cross-sectional questionnaire was devised to seek retrospective information on mothers’ breastfeeding practices of their youngest child (index child), and to explore factors influencing breastfeeding initiation and duration. Mothers were asked to provide information on whether they had breastfed their index child. If they had, the total length of time that the index child had been breastfed was documented. Variables related to maternal socio-demographics, biomedical and behavioural information, social support and influence, and attitudes, which have potential influence on breastfeeding initiation and/or duration were included in the questionnaire and listed in Table 1 and Table 6 in Appendix. The questionnaire was devised on the basis of an extensive review of the migration literature of breastfeeding [12, 18, 39–41]. Some breastfeeding attitude questions were adopted from the Iowa Infant Feeding Attitude Scale (IIFAS) [40]. Some questions were investigator-created. The questionnaire took approximately 30 min to complete. It was reviewed for content validity, reliability and cultural appropriateness by two public health researchers who had extensive research experience in breastfeeding, and a Chinese medical doctor. It was translated into Chinese and blind back-translated to check the accuracy of the translation [42]. The questionnaire (Chinese) was pilot tested on 20 Chinese mothers in Ireland to assess clarity, redundancy and language adequacy.

**Table 1:** Socio-demographic characteristic of the samples (Phase 1)

	Total population	CMC	CMI	*P* value
	(n = 322)	(n = 47)	(n = 275)	
	No. (%)	No. (%)	No. (%)	
Mother’s age at time of childbirth (years)				< 0.001
20–25	108 (34.4)	27 (60.0)	81 (30.1)	
26–30	121 (38.5)	10 (22.2)	111 (41.3)	
> 30	85 (27.1)	8 (17.8)	77 (28.6)	
Marital status				0.265
Married	275 (85.4)	43 (91.5)	232 (84.4)	
Single/Divorced/Widow	47 (14.6)	4 (8.5)	43 (15.6)	
Mothers’ education level				1.00
Primary/Secondary	156 (48.4)	23 (48.9)	133 (48.4)	
Tertiary	166 (51.6)	24 (51.1)	142 (51.6)	
Mother’s birthplace				0.656
Mainland China	273 (85.3)	39 (83.0)	234 (85.7)	
Hong Kong	47 (14.7)	8 (17.0)	39 (14.3)	
Maternal length of Irish residency at time of interview (years)				< 0.001
< =5	98 (30.4)	29 (61.7)	69 (25.1)	
> 5–10	180 (55.9)	17 (36.2)	163 (59.3)	
> 10	44 (13.7)	1 (2.1)	43 (15.6)	
Child’s order				< 0.001
1	216 (67.1)	45 (95.7)	171 (62.2)	
2/3/4	106 (32.9)	2 (4.3)	104 (37.8)	
Child’s gender				0.636
Male	168 (52.7)	23 (48.9)	145 (53.3)	
Female	151 (47.3)	24 (51.1)	127 (46.7)	
Mother herself having been breastfed as a baby				0.115
Yes	276 (85.7)	44 (93.6)	232 (84.4)	
No/Don’t know	46 (14.3)	3 (6.4)	43 (15.6)	
Husband’s nationality				0.800
Chinese	280 (88.3)	40 (90.9)	240 (87.9)	
Not Chinese	37 (11.7)	4 (9.1)	33 (12.2)	
Husband‘s education level				0.332
Primary/Secondary	185	23 (51.1)	162 (59.1)	
Tertiary	134	22 (48.9)	112 (40.9)	
Mother’s current occupation^a^				
Self-employed/ Professional work			62 (22.9)	
Non-professional work			89 (32.8)	
Housewife			120 (44.3)	
Mother’s current accommodation^a^				
Rented			189 (69.0)	
Family’s own property			85 (31.0)	
Mother’s self-perceived level of English comprehension^a^				
Very good/good			142 (51.6)	
Not good/Not at all			133 (48.4)	
Husband‘s current occupation^a^				
Self-employed/ Professional work			87 (32.2)	
Non-professional work			175 (64.8)	
Unemployed			8 (3.0)	
Family income (Euro, before tax)^a^				
< 15,000			40 (14.9)	
15,000030,000			134 (50.0)	
> 30,000			94 (35.1)	
	Mean ± S.D.	Mean ± S.D.	Mean ± S.D.	
Child’s age at time of the interview	5.22 ± 4.68	11.72 ± 5.65	4.11 ± 3.46	< 0.001

*CMC* Chinese mother gave birth in China, *CMI* Chinese mother gave birth in Ireland

Columns where the numbers do not add up to the specific n reflect missing values for this column

*P* value was obtained from Pearson Chi-square statistics (categorical variables) or Independent sample t-test (continuous variables) to detect the differences between CMC and CMI. *P* < 0.05 was considered as significant different

Mainland China included the provinces of Anhui, Hebei, Heilongjiang, Henan, Hubei, Jiangsu, Jilin, Liaoning, Shandong, Shanxi; Fujian, Guangdong, Jiangxi, Sichuang, Yunnan, Zhejiang; Xinjiang Uygur Autonomous Region; Guangxi Zhuang Autonomous Region; and the municipality of Beijing, Tianjin, Chongqing, and Shanghai

Hong Kong indicated Hong Kong Special Administrative Regions of China. In this study, one subject from Macau was grouped into this category

^a^These variables were only investigated among CMI but not the whole study population

#### Phase 2

In Phase 2, a brief self-administered questionnaire was used to collect socio-demographic information, and to assess whether mothers had breastfed their children born in Ireland (and any previous children born in China).

### Data analyses

#### Phase 1

Phase 1 (quantitative study) was initially designed to describe breastfeeding initiation and duration among Chinese mothers living in Ireland (i.e. ‘all participants’). Upon the completion of data collection, sample size calculation based on Daly and Bourke [43] found that there were adequate number of participants to perform group comparison and subgroup analyses. ‘All participants’ were further separated into two subsamples: Chinese mothers who gave birth in China (CMC) and Chinese mothers who gave birth in Ireland (CMI). Mothers who had given birth in both China and Ireland (*n* = 25) were classified as CMI as all of them gave birth to the index child in Ireland. Breastfeeding practices of CMC and CMI were compared, as these two groups were likely to share some common characteristics, which made them more comparable in understanding whether the variation in breastfeeding rates among Chinese in China versus Ireland was due to migration or other factor(s). Moreover, recruiting participants in one place (i.e. Ireland) was more convenient than recruiting a reference group of Chinese mothers who were still living in China. It was estimated that the size of groups had 80% power to detect a difference of 10.4% in breastfeeding initiation rate. For the socio-demographic data, mean and standard deviation (SD) were computed for continuous variables and frequency of occurrence (percentage) was computed for categorical variables. Univariate analyses (Pearson’s Chi-square tests for categorical variables and independent sample t-tests for continuous variables) were conducted to assess the differences between CMC and CMI. Pearson’s Chi-square tests were conducted to assess the differences of the prevalence of breastfeeding from birth to 12 months between CMC and CMI.

Determinants of breastfeeding were conducted among ‘all participants’ and CMI, respectively. ‘Breastfeeding initiation’ was defined as a dichotomous variable (Yes/No). Since the median duration of breastfeeding for CMI was 4.0 months, ‘breastfeeding duration of 4 months’ was selected as the cut-off point in defining the dichotomous variable of breastfeeding duration. Univariate logistic regression analyses were performed to test each variable (Table 1 and Table 6 in Appendix) individually and its potential association with two outcomes, i.e. breastfeeding initiation and duration. Selected socio-demographic variables including couple’s current occupations, current accommodation and family income were only investigated among CMI but not CMC, because these variables reflected mothers’ status in Ireland at time of the study and had no influence on CMC’s childbearing status in China. To adjust for potential confounders, multivariate logistic regression analyses were conducted, by entering variables having a significant level of *P* < 0.15 in the univariate analyses (see Table 7 and 8 in Appendix for details). Child’s current age was also entered in each model to control for any time-related differences in breastfeeding. For ‘all participants’, two socio-demographic models were employed to assess the independent effect of child’s birthplace on breastfeeding initiation and duration, respectively. For CMI, a series of models were applied to explore the determinants of breastfeeding, including ‘Socio-demographic & Social support and influence Model’, ‘Social-demographic & Behavioural Model’, ‘Socio-demographic & Attitudinal Model’, and ‘Full Model’ (which included all variables having a significant association with breastfeeding initiation/duration in the univariate analysis and listed in Table 7 and 8 in Appendix). Different models were generated to assess the individual and combined impact of modifiable factors (e.g. attitude, behaviour) of breastfeeding. Socio-demographic variables were controlled in each model as they were considered as unmodifiable factors of breastfeeding and might be associated with attitudes and behaviours. The overall fit of each model was accessed by the log-likelihood statistics (−2LL). A small -2LL indicates a good fit of the model [44]. Quantitative data analyses were conducted with SPSS (version 20). Statistically significant was deemed as *P* < 0.05.

#### Phase 2

Thematic content analysis was performed by QZ in Phase 2, following the guidelines recommended by Morse & Field [45], with the application of the software NVivo 8.0 (QSR International; Melbourne, VIC, Australia). Open coding was conducted by reading the transcripts sentence-by-sentence. Codes were then organized into categories and integrated into themes, with the assistance of a coding tree. Themes were derived from the data. Field notes were reviewed with the transcripts during the process. Preliminary results in the form of a two pages’ summary were sent to 11 randomly selected participants for respondent validation. All participants gave confirmatory feedback. Qualitative results were further translated and interpreted in English by the author QZ.

#### Integration of the quantitative and qualitative data

In addition to the connection in data collection stage, the quantitative and qualitative data were further integrated after independent analyses. The quantitative results illustrated the prevalence and determinants of breastfeeding initiation and duration, and the qualitative results provided an in-depth explanation of the phenomenon and barriers to breastfeeding. Both results were integrated in the discussion, and shared equal priority [38, 46].

## Results

### Characteristics of the phase 1 participants

A total of 343 questionnaires were collected. With an exclusion of those who had not delivered their baby in China or Ireland, the final sample population was 322, including 47 CMC and 275 CMI. Table 1 illustrated the socio-demographic characteristic of the participants. The majority of mothers was married, born in Mainland China, primiparous, and had received tertiary education. CMI were older at time of index birth, less likely to be primiparous, and had been in Ireland for a longer duration, in comparison with CMC. The age of the index child at time of survey for overall sample, for CMC and CMI were 5.22, 11.72 and 4.11 years, respectively. No CMC were still breastfeeding the index child at the time of migration to Ireland (data not shown).

### Breastfeeding rates between CMC and CMI (results of phase 1)

**Fig. 1:**
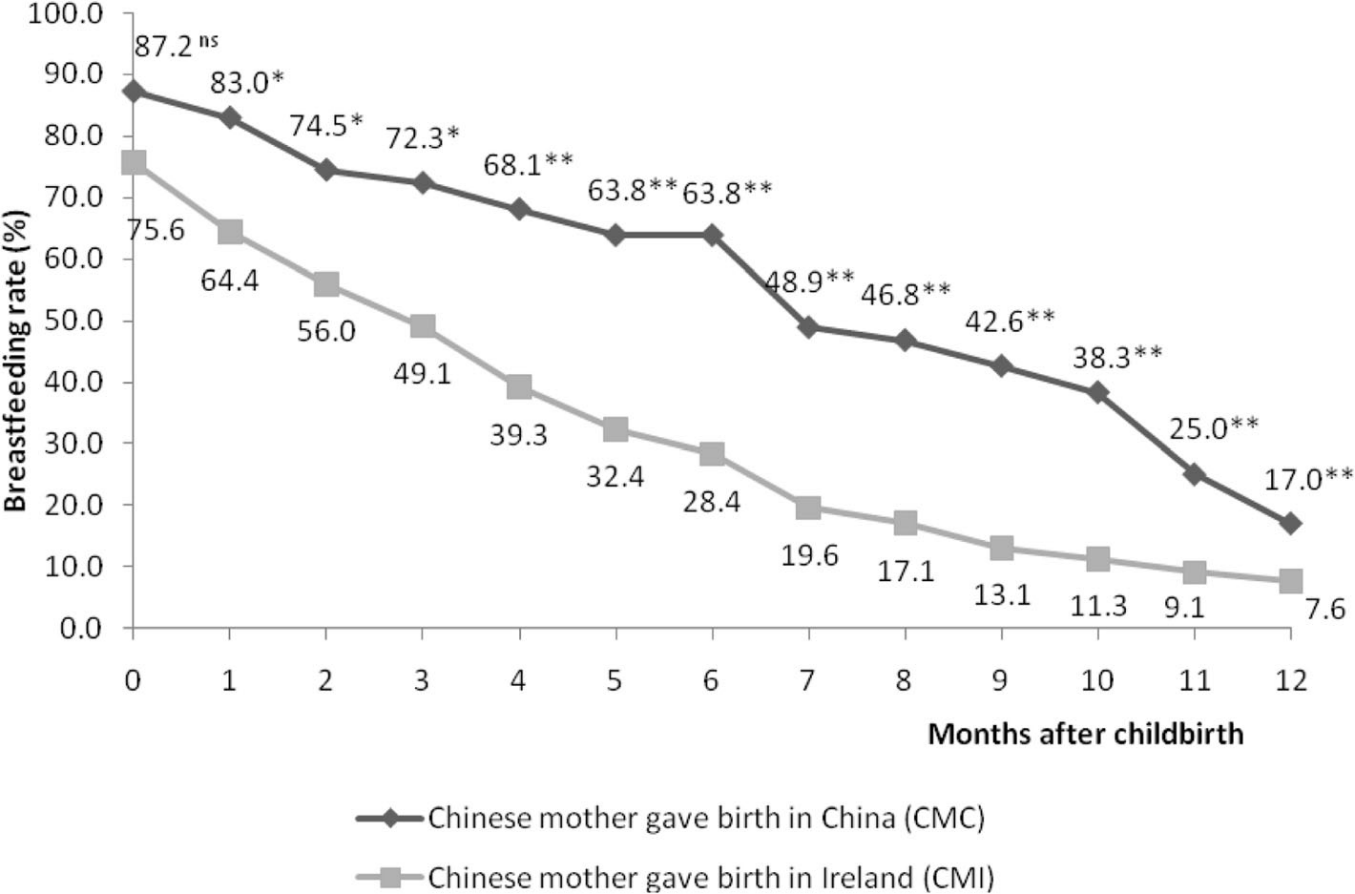
Breastfeeding prevalence between CMC (*n* = 47) and CMI (*n* = 275) from birth to 12 months. *P < 0.05; **P < 0.001; ns: no significant difference. Significance of relationships was calculated by Pearson Chi-square statistics

Figure 1 illustrates the ‘any breastfeeding’ rates between CMC and CMI, from childbirth to 12 months of age. Initially, 41 out of 47 CMC (87.2%) and 208 out of 275 CMI (75.6%) breastfed their child. Rates of CMI dropped to 49.1% at 3 months and 28.4% at 6 months whereas the rate among CMC at 6 months remained above 60%. At 12 months, breastfeeding rates of CMC and CMI fell to 17 and 7.6%, respectively. Breastfeeding rates of CMI were significant lower than that of CMC from 1 month to 3 months (*P* < 0.05). Even more distinct differences (i.e. CMI had lower breastfeeding rates than CMC) were found from 4 months to 12 months (*P* < 0.001).

### Determinants of breastfeeding initiation and duration among ‘all participants’ (results of phase 1)

Among ‘all participants’, child’s birthplace had no significant independent effect on breastfeeding initiation, but was the strongest socio-demographic predictor for longer duration of breastfeeding. CMC was 4.64 times more likely to breastfeed for at least 4 months, compared to CMI (adjusted OR = 4.64, 95% CI: 1.78–12.06, *P* < 0.05) (Table 2). Mothers with higher education level and herself had been breastfed were determinants of a longer duration of breastfeeding. In addition, univariate analyses indicated that mothers who were born in Hong Kong were less likely to initiate breastfeeding and breastfeed for 4 months and above, in comparison to mothers born in Mainland China (Table 2).

**Table 2:** Socio-demographic determinants of breastfeeding initiation and duration among all participants (n = 322, Phase 1)^§^

	Initiated breastfeeding		Odds Ratio (95% CI)		Breastfed ≥4 months		Odds Ratio (95% CI)	
	Yes (%)	No (%)	Unadjusted	Adjusted^a^	Yes (%)	No (%)	Unadjusted	Adjusted^b^
Child’s birthplace								
China	41 (87.2)	6 (12.8)	2.20 (0.90–5.41)	2.48 (0.74–8.28)	32 (68.1)	15 (31.9)	3.35 (1.73–6.48)*	4.64 (1.78–12.06)*
Ireland	208 (75.6)	67 (24.4)	1	1	107 (38.9)	168 (61.1)	1	1
Maternal birthplace								
Mainland China	220 (80.3)	54 (19.7)	2.88 (1.49–5.54)*	1.41 (0.57–3.53)	129 (47.1)	145 (52.9)	3.20 (1.53–6.71)*	1.48 (0.59–3.68)
Hong Kong	27 (58.7)	19 (41.3)	1	1	10 (21.7)	36 (78.3)	1	1
Maternal education level								
Tertiary	139 (83.7)	27 (16.3)	2.15 (1.26–3.68)*	1.65 (0.87–3.14)	91 (54.8)	75 (45.2)	2.73 (1.73–4.31)*	2.08 (1.20–3.62)*
Primary/Secondary	110 (70.5)	46 (29.5)	1	1	48 (30.8)	108 (69.2)	1	1
Mother has been breastfed								
Yes	222 (80.4)	54 (19.6)	2.89 (1.50–5.59)*	1.78 (0.81–3.91)	131 (47.5)	145 (52.5)	4.29 (1.93–9.53)*	2.67 (1.10–6.44)*
No/Don’t know	27 (58.7)	19 (41.3)	1	1	8 (17.4)	38 (82.6)	1	1
Husband education level								
Tertiary	113 (84.3)	21 (15.7)	2.05 (1.62–3.61)*	1.33 (0.67–2.61)	75 (56.0)	59 (44.0)	2.46 (1.56–3.89)*	1.53 (0.89–2.64)
Primary/Secondary	134 (72.4)	51 (27.6)	1	1	63 (34.1)	122 (65.9)	1	1
Parity								
Primiparous	177 (81.9)	39 (18.1)	2.14 (1.26–3.66)*	1.41 (0.76–2.64)	103 (47.7)	113 (52.3)	1.77 (1.09–2.87)*	0.93 (0.53–1.66)
Multiparous	72 (67.9)	34 (32.1)	1	1	36 (34.0)	70 (66.0)	1	1
	Mean ± SD	Mean ± SD			Mean ± SD	Mean ± SD		
Child‘s current age (years)	5.1 ± 4.70	5.7 ± 4.62	0.97 (0.92–1.03)	0.98 (0.90–1.07)	5.2 ± 5.02	5.2 ± 4.42	1.00 (0.95–1.05)	0.96 (0.89–1.03)
Maternal age at childbirth (years)	27.8 ± 4.39	28.5 ± 4.92	0.97 (0.91–1.02)^c^	na	28.1 ± 4.61	27.9 ± 4.44	1.01 (0.97–1.07)^d^	na

*P < 0.05. ^a^ -2LL = 311.088, df = 7. ^b^ -2LL = 380.546, df = 7. ^c^*P* = 0.240. ^d^*P* = 0.593. na: not available

^§^This table illustrates two socio-demographic models that assess the independent effect of child’s birthplace on breastfeeding initiation and duration. First, each independent variable was assessed by univariate analyses individually. Unadjusted Odds Ratios (ORs) and P values were obtained. Second, variables having significant effect (*P* < 0.15) on the outcome were entered together in a logistic regression model. Child’s birthplace was entered in the model regardless of its significance. Child’s current age was also entered in the model regardless of its significance, to control for any time-related differences in breastfeeding. Adjusted ORs and *P* values were obtained. For continuous variables, it was indicated that when age increased one year, the odds of initiating breastfeeding / breastfeeding≥4 months would increase (OR-1) times

### Determinants of breastfeeding initiation and duration among CMI (results of phase 1)

Table 3 and Table 4 display regression models of breastfeeding initiation and duration among CMI, respectively. As found in the univariate (Table 7 and 8 in Appendix) and multivariate (Table 3 and Table 4) analyses, attitudinal factors played the most important role in determining breastfeeding initiation and duration, in comparison to socio-demographic, behavioural, and social support and influence factors.

**Table 3:** Significant determinants of breastfeeding initiation of CMI, after adjustment for potential confounders (n = 275, Phase 1)

	Socio-demographic & Social support and influence Model^a^		Socio-demographic & Behavioural Model^b^		Socio-demographic & Attitudinal Model^c^		Full Model^d^	
	Adjusted OR (95% CI)	*P* value	Adjusted OR (95% CI)	*P* value	Adjusted OR (95% CI)	*P* value	Adjusted OR (95% CI)	*P* value
Past breastfeeding experience: had given birth but not breastfed to any child (ren) vs. had given birth and breastfed			0.15 (0.05–0.45)	0.001				
Maternal occupation: Self-employed/Professional work *v.s*. housewife	3.68 (1.26–10.76)	0.017	2.89 (1.08–7.69)	0.034				
Feeding information obtained from the antenatal classes	2.48 (1.09–5.63)	0.030					3.14 (1.05–9.41)	0.041
Health professionals’ attitude influenced mother’s feeding choice	4.54 (1.67–12.31)	0.003						
Feeding decision was made:								
Before pregnancy vs. After the child was born					20.92 (6.85–63.93)	< 0.001	19.06 (5.87–61.91)	< 0.001
During pregnancy vs. After the child was born					7.75 (2.69–22.33)	< 0.001	6.12 (2.05–18.25)	0.001
Attitude towards colostrum: important for the baby					4.33 (1.65–11.31)	0.003	3.25 (1.14–9.27)	0.028
Disagree with ‘I don‘t like breastfeeding’					7.10 (2.76–18.24)	< 0.001	7.41 (2.56–21.45)	< 0.001

‘Full Model’ included all variables (socio-demographic, behavioural, social support and influence, and attitudinal) which have an association with breastfeeding initiation (*P* < 0.15) among CMI, according to the univariate logistic regression analyses

^a^ -2LL = 246.458, df = 17. Non-significant variables that were included but not depicted in this model: maternal length of Irish residency at time of interview, child’s age, maternal birthplace, couple’s education level, parity, accommodation, husband’s occupation, child’s gender, mother herself had been breastfed, infant feeding information obtained from the internet, mother’s own mother, and feeding choice was influenced by mother’s own mother’s attitude

^b^ -2LL = 253.501, df = 14. Non-significant variables that were included but not depicted in this model: maternal length of Irish residency at time of interview, child’s age, maternal birthplace, mother’s education level, mother herself had been breastfed, husband’s education, husband’s occupation, parity, accommodation, child’s gender, and the practice of bed sharing

^c^ -2LL = 185.551, df = 20. Non-significant variables that were included but not depicted in this model: maternal length of Irish residency at time of interview, child’s age, maternal birthplace, couple’s education level, couple’s occupation, mother had been breastfed as a child, parity, accommodation, child’s gender, agreement/disagreement with ‘formula feeding is more convenient than breastfeeding’, ‘I would feel embarrassed if someone saw me breastfeeding’, ‘Some traditional Chinese food can help to improve milk production’, and maternal awareness of the advantages of breastfeeding

^d^ -2LL = 168.592, df = 27. Non-significant variables that were included but not depicted in this model: maternal length of Irish residency at time of interview, child’s age, maternal birthplace, couple’s education level, couple’s occupation, mother had been breastfed as a child, parity, accommodation, child’s gender, the practice of bed sharing, mother’s past breastfeeding experience, infant feeding information obtained from internet and mother’s own mother, feeding choice was influenced by mother’s own mother’s attitude and health professionals’ attitude, agreement/disagreement with ‘formula feeding is more convenient than breastfeeding’, ‘I would feel embarrassed if someone saw me breastfeeding’, ‘Some traditional Chinese food can help to improve milk production’, and maternal awareness of the advantages of breastfeeding

**Table 4:** Significant determinants of breastfeeding duration ≥4 months of CMI, after adjustment for potential confounders (n = 275, Phase 1)

	Socio-demographic & Social support and influence Model^a^		Social-demographic & Behavioural model^b^		Social-demographic & Attitudinal model^c^		Full Model^d^	
	Adjusted OR (95% CI)	*P* value	Adjusted OR (95% CI)	*P* value	Adjusted OR (95% CI)	*P* value	Adjusted OR (95% CI)	*P* value
Mother had been breastfed as a child	3.22 (1.21–8.56)	0.019	3.81 (1.22–11.88)	0.021				
Feeding information obtained from mothers’ own mother	1.97 (1.08–3.59)	0.027						
Child was looked after by maternal grandmother							4.53 (1.21–16.92)	0.025
Child was looked after by paternal grandmother	0.17 (0.05–0.61)	0.007						
Mother bed shared with the baby			3.10 (1.46–6.62)	0.003			3.27 (1.32–8.11)	0.011
Breastfed within 1st hour after childbirth			2.14 (1.07–4.25)	0.030				
Formula was introduced at least 1 week after the child was born			2.81 (1.37–5.77)	0.005				
Child was fed on demand			2.68 (1.42–5.05)	0.002			2.89 (1.36–6.13)	0.006
Mother consumed cultural postpartum diet			2.88 (1.46–5.66)	0.002				
Feeding decision was made:								
Before pregnancy vs. After the child was born					4.95 (1.17–20.90)	0.030		
During pregnancy vs. After the child was born					4.61 (1.04–20.44)	0.044		
Planned breastfeeding duration: ≥ 4 months vs. < 4 months					9.66 (4.18–22.31)	< 0.001	10.09 (3.83–26.61)	< 0.001

‘Full Model’ included all variables (socio-demographic, behavioural, social support and influence, and attitudinal) which have an association with breastfeeding duration (*P* < 0.15) among CMI, according to the univariate logistic regression analyses

^a^ -2LL = 305.873, df = 16. Non-significant variables that were included but not depicted in this model: mother’s age at time of childbirth, maternal length of Irish residency at time of interview, child’s age, maternal birthplace, mother’s education, husband’s education level, couple’s occupation, husband’s nationality, infant feeding information obtained from antenatal classes, and child was looked after by mother’s own mother

^b^ -2LL = 253.812, df = 19. Non-significant variables that were included but not depicted in this model: mother’s age at time of childbirth, maternal length of Irish residency at time of interview, child’s age, maternal birthplace, husband’s education level, couple’s occupation, husband’s nationality, and mother’ previous breastfeeding experience

^c^ -2LL = 236.114, df = 19. Non-significant variables that were included but not depicted in this model: mother’s age at time of childbirth, maternal length of Irish residency at time of interview, child’s age, maternal birthplace, couple’s education level, couple’s occupation, mother had been breastfed as a child, husband’s nationality, agreement/disagreement with ‘I don‘t like breastfeeding’, ‘formula feeding is more convenient than breastfeeding’, and ‘I would feel embarrassed if someone saw me breastfeeding’, as well as maternal awareness of the advantages of breastfeeding

^d^ -2LL = 200.045, df = 30. Non-significant variables that were included but not depicted in this model: mother’s age at time of childbirth, maternal length of Irish residency at time of interview, child’s age, maternal birthplace, couple’s education level, couple’s occupation, mother had been breastfed as a child, husband’s nationality, infant feeding information obtained from mother’s own mother and antenatal classes, child was looked after by mother’s mother-in-law, timing of the introduction of breast milk and infant formula, mother consumed cultural postpartum diet, past breastfeeding experience, timing of the feeding decision made, agreement/disagreement with ‘I don‘t like breastfeeding’, ‘formula feeding is more convenient than breastfeeding’, and ‘I would feel embarrassed if someone saw me breastfeeding’, as well as maternal awareness of the advantages of breastfeeding

In the ‘Full Model’ that predicts initiation, mothers who disagreed with ‘I don‘t like breastfeeding’ and who considered colostrum important for the baby were 7.41 and 3.25 times more likely to initiate breastfeeding than their peers, respectively. Mothers who made their feeding decision before pregnancy (adjusted OR = 19.06, 95% CI: 5.87–61.96, *P* < 0.001) or during pregnancy (adjusted OR = 6.12, 95% CI: 2.05–18.25, *P* = 0.001) were more likely to initiate breastfeeding than those who chose their feeding method after the child was born. Regarding to the social support and influence, mothers whose feeding choice was influenced by the health care professionals (adjusted OR = 4.54, 95% CI: 1.67–12.31, *P* = 0.003), who obtained feeding information from the antenatal classes (adjusted OR = 2.48, 95% CI: 1.09–5.63, *P* = 0.030), and who were self-employed or had professional work (adjusted OR = 3.68, 95% CI: 1.26–10.76, *P* = 0.017) were positively associated with breastfeeding initiation. Among multiparous mothers, who had previous breastfeeding experience were more likely than those who had never breastfed to initiate breastfeeding, according to the ‘Socio-demographic & Behavioural Model’ (Table 3).

In the ‘Full Model’ that predicts duration, planned breastfeeding duration of at least 4 months were positively associated with an actual breastfeeding duration of 4 months or more. Mothers who bed-shared with the child (adjusted OR = 3.27, 95% CI: 1.32–8.11, *P* = 0.011), who fed the child on demand (adjusted OR = 2.89, 95% CI: 1.36–6.13, *P* = 0.006), and whose child being looked after by maternal grandma during the first few months after birth (adjusted OR = 4.53, 95% CI: 1.21–16.92, *P* = 0.025) were positively associated with an actual breastfeeding duration of 4 months or more. In addition to the behaviours of bed-sharing and on-demand feeding, mother who consumed cultural postpartum diet, who breastfed within the first hour after childbirth, and who introduced infant formula at least 1 week after childbirth were more likely to breastfeed for at least 4 months, according to the ‘Socio-demographic & Behavioural Model’ (Table 4).

### Influence of living in Ireland on breastfeeding practices (results of phase 2)

Seven focus groups were conducted, lasting 40 to 70 min, and 33 Chinese mothers were interviewed. Participants were averagely 35.1 years old, and had been living in Ireland for 8.5 years. Over 75% of the participants had breastfed at least one child (Table 5). The youngest child born in Ireland at time of the focus group was averagely 5.1 years old (data not shown). Content analyses revealed the main reasons for a short duration of breastfeeding among Chinese mothers who gave birth in Ireland, which were detailed below.

**Table 5:** Characteristics and breastfeeding rates of the focus group participants in Phase 2 (n = 33)

Characteristics	Mean ± SD or n (%)
Maternal current age (years)	35.1 ± 6.82
Mother’s length of Irish residency (years)	8.5 ± 4.50
Mother’s birthplace	
Liaoning province (located in northern China)	8 (24.2)
Fujian province (located in southern China)	10 (30.3)
Other provinces	6 (18.2)
Hong Kong	9 (27.3)
Marital status	
Married	31 (93.9)
Single	2 (6.1)
Maternal education level	
Primary school	1 (3.0)
Secondary school	10 (30.3)
Training school	16 (48.5)
Third level	6 (18.2)
Maternal current occupation	
Housewife	18 (54.5)
Non-professional jobs	15 (45.5)
Maternal delivery place^a^	
Ireland	19 (57.6)
Ireland and China	14 (42.4)
Has mother ever breastfed?	
Yes	25 (75.8)
No	8 (24.2)

^a^Information about countries where mother had given birth to all her children (i.e. the index child plus other children)

#### Cultural conflicts

Participants believed that breastfeeding is beneficial to child health; however, breast milk quantity and quality were their major concerns. Participants believed that various soups, such as pig feet and peanut soup, papaya and fish soup, and shellfish soup, should be consumed to boost breast milk supply duration the confinement period (the first month after childbirth). The practices of drinking tap and icy water, bathing and hair washing within a few days after childbirth were perceived to impair maternal health, resulting in poor breast milk quality. Participants believed that breastfeeding should stop if breast milk becomes watery, as ‘watery’ breast milk was perceived to be in poor milk quality by the participants.

Without an understanding of the Chinese culture, health care professionals in Ireland gave advice that was against the Chinese cultural restrictions. To avoid being deemed as difficult, participants followed the Irish rituals (e.g. took a shower soon after childbirth) during their stay in hospital. Cultural conflicts in hospital practices made participants worried, upset, anxious or even depressed, and thereby considered their breast milk insufficient and of poor quality.

*“When I phone China, my mother reminded me to avoid cold and wind. But here in Ireland, I had to follow the doctors’ advice and the Irish ritual. Otherwise I would be considered to be very strange (by staffs in the hospital)”.*

*“She (public health nurse) said you would have enough milk if you drank more water. But we are Chinese people; we have to have soup, like fish soup.”*

#### A lack of family support

During the postpartum period, only one participant indicated that her own mother had come to Ireland to offer family support. Most participants were living in a nuclear family in Ireland. Husband, who was often the only source of family support, was also the main source of financial support and thus had to work. Without adequate assistance from family members in housework, maternal diet preparation and child minding, our participants failed to ensure the cultural confinement practices (e.g., consuming a traditional diet to boot breast milk production, not doing housework or touching cold water, not going outside). Considerable concern about breast milk supply was thereby triggered. A participant who had breastfed her children for no more than 1 month said:

*“Mothers in China may receive more care and support from family; therefore they recover better and have sufficient breast milk. They can breastfeed for a longer duration (than us in Ireland). For most of us in Ireland, our parents were far away, and our husband had to work. Nobody was available to look after us during the confinement period.”*

#### Language barriers

Owing to their English language barriers, participants indicated difficulties to access to breastfeeding education and information in Ireland, in particular from the health care professionals. When confronted with breastfeeding difficulties, participants did not receive adequate support or advice from the health care professionals. Breastfeeding had to be stopped as difficulties had not been overcome timely and appropriately.

*“I was invited to attend the training classes for breastfeeding in Ireland. I did not attend because my English was not good.”*

#### Low socioeconomic status

All participants who were working (45.5%) had non-professional occupations in Ireland (Table 5). For economic reason, some participants had to work for long hours, and returned to work early after childbirth. Four participants indicated that they sent the child back to China to be looked after by other family members about 3 months after childbirth. These participants indicated a prenatal intention to abandon breastfeeding or breastfeed for a very short period.

*“We came to Ireland for economic reason. It was not good to have our baby beside us. We had to work. If we took good care of the baby, we had to stop working, which was impossible.”*

*“Breastfeeding is the best. However, if you want to send the child back China, you have to be aware of the difficulties of weaning the baby once you start.”*

#### Maternal preference for the infant formula in Ireland

Infant formula in Ireland was widely considered as good quality, safe, and of reasonable price, in comparison to that produced in China. Use of infant formula was prevalent among the participants, resulting in abandonment or a shorter duration of breastfeeding.

*“Breastfeeding is very good. But I have not seen any disadvantages of formula feeding. I found that children fed with infant formula in Ireland were growing well and fast.”*

## Discussion

### The participants

This is the first and only study on breastfeeding initiation and duration among the Chinese in Ireland. The quantitative data was collected among a convenience sample of Chinese immigrants in Ireland, representing 7% of the Chinese female population aged 20–54 years in Ireland (*n* = 4660, data from the Irish census 2006) [33]. A higher proportion of our participants were living in Dublin metropolitan area (approximately 80% [data not shown] *v.s.* 67%), married (85.4% vs. 65%), and had a tertiary education level (51.6% vs. 28%), in comparison to the corresponding figures in census 2006 [33]. Phase 1 and Phase 2 include participants born in the northern, southern, and the special administrative region of China; while no profile about the origin of the Chinese immigrants in Ireland has been reported.

### Breastfeeding initiation and duration

Migration to Ireland appears to be related to breastfeeding duration but not initiation of the Chinese immigrants in this study. Initiation rates for both CMC and CMI are close to the Chinese national target rate of 85% in 2010 [15], and much higher than that of the Irish nationals (44–56% reported in different studies) [3, 24–26, 28]. The initiation rate of CMI is similar to that of Chinese immigrants in Australia (79–86%) [12, 20] and in Canada (78%) [47] (studies conducted in late 1990s to early 2000s), but higher than that of Chinese immigrants in the UK (2%) [16] and in the US (10–30%) [17] (studies undertaken 15 to 20 years prior to our data collection). On one hand, higher initiation rate observed in our study in comparison to that reported in the UK and the US might imply an overall improvement in breastfeeding initiation rate worldwide. On the other hand, the low breastfeeding initiation rates reported in the UK and US studies might be because the immigrant Chinese mothers participated in those two studies were dominated by Hong Kong mothers who were less likely to initiate breastfeeding than mothers from Mainland China.

Breastfeeding duration of CMI was significantly shorter than that of CMC and also shorter than the mean duration (7–9 months, reported by a review summarizing studies conducted between 1994 and 2004) in the majority of cities in China [15]. Short breastfeeding duration among CMI concurs with a number of Chinese migration studies [18, 47, 48] and other Asian migration studies [14, 49, 50] in Western countries. The consistency in these findings suggests the important influence of migration on breastfeeding duration. The influences of cultural conflicts on postnatal practices, a lack of support from extended family members, language difficulties in communicating with health professionals on breastfeeding practices highlighted in this study have been reported among other Asian immigrants in Western countries [11, 49, 51–54].

### The influence of migration on breastfeeding practices

Our quantitative data which shows an association between the consumption of traditional postpartum diet and breastfeeding duration among CMI, could be explained by the cultural belief of the efficacy of diet on breast milk production, revealed in our qualitative analyses. However, the cultural belief is not supported by Western biomedical wisdom, in which breast milk production is merely considered to be dependent on hormonal controls and frequent emptying of the breast [55]. The Chinese immigrants who have difficulties in consuming the traditional diet should be educated on alternative ways to enhance breast milk production, such as frequent sucking recommended by the WHO [6].

Health care professionals are found to be important persons in influencing breastfeeding initiation in our quantitative study. However, consistent with the Chinese migration literature [56–58], our qualitative finding demonstrates that the health care professionals in Ireland are not aware of the cultural beliefs of their clients. Moreover, in keeping with the influence of the Confucian-based Chinese culture that emphasizes the importance of maintaining harmonious relationships with others [59], Chinese mothers in our study adhered to health care professionals’ advice, even when it was contradictory to their own cultural norm. As a result, it is suggested that health care professionals in Ireland pay particular attention to the cultural beliefs of ethnic groups, and offer culturally sensitive support and advice to the immigrants.

Language barrier is a common problem among immigrants globally [53, 54], including our study participants. In addition to qualitative quotes, our quantitative data illustrates that very few CMI obtained infant feeding information from antenatal classes (Table 6 in Appendix). Language specific breastfeeding promotion and education is therefore recommended. The provision of Chinese-speaking nurses and Chinese-speaking prenatal courses [13], and the distribution of prenatal manuals written in Chinese in the health centres [13] which have found to be successfully in improving breastfeeding duration among Chinese immigrants in Canada, might be adopted in Ireland.

In the migration literature, an absence of assistance from extended family has extensively been found to contribute to mothers’ infrequent consumption or unavailability of the postnatal diet, starting housework earlier than expected, and fatigue [56, 60, 61], which are causes to breastfeeding cessation. Our results could be explained by the Chinese culture that postnatal support is often offered by a female family member but not husband [21, 58, 61–63]. Our quantitative results indicate that breastfeeding duration of CMI was associated with the consumption of cultural postpartum diet, and support from the maternal grandmother in child minding. Such quantitative results, together with our relevant qualitative quotes, suggest the negative influence of a lack of family support on breastfeeding duration. In recognition of the importance of family support to breastfeeding continuation, maternal grandmothers’ coming to Ireland to provide postnatal support to new mothers might be considered. Realistic alternatives, such as facilitation of connections within the Chinese community living in Ireland, might be explored if extended family visits are not possible.

To our knowledge, this is the first study reporting the issue of sending immigrants’ children born in the host country back to their home country, as a barrier to breastfeeding initiation and duration. Future studies among Chinese immigrants in other countries might take this issue into account. Notably, patterns of migration vary between countries and time periods. The Chinese labourers have a history of settling down in Canada, US and Australia for over 150 years [64]. In the recent 30 years, the Chinese immigrants over the world are dominated by students, who are likely to return to China after receiving higher education and some working experience [64]. The Chinese immigrants in Ireland in the recent decades are mainly students and migrant workers [33], who do not have sufficient time for child caring. We speculate that sending the child back to China is a unique issue for non-permanent Chinese immigrants in recent decades.

Favourable attitudes towards infant formula on the Western market found in this study may be unique to the Chinese population, because the safety of infant formula made in China has been of great public concern [65]. In 2008, ‘melamine-contaminated formula milk’ appeared on the Chinese market, harming a lot of infants’ health [66]. Moreover, our focus group participants did not see any disadvantages of formula feeding. Introduction of infant formula within 1 week postpartum, a practice independently related to a shorter duration of breastfeeding of CMI, is prevalent among the Chinese immigrants in this study. Education on the appropriate timing for infant formula introduction should be given to Chinese immigrants in Ireland.

Our quantitative findings among CMI are in consistent with the literature that maternal antenatal feeding intention is a strong determinant of breastfeeding initiation and duration [26, 67, 68]. Our study also reveals that feeding information obtained from the antenatal classes is associated with breastfeeding initiation. Efforts to increase antenatal class attendance with particular emphasis on methods to increase breast milk production and optimal breastfeeding duration might be considered in Ireland. Our findings also add to the literature that attitudinal factors appear to be more important than socio-demographic factors in determining breastfeeding initiation and duration [69–71], suggesting a possible means to promoting breastfeeding among Chinese immigrants, a population who are often disadvantaged by their lack of family support. Behavioural factors associated with a longer duration of breastfeeding identified in our study (e.g. breastfeeding within the first hour after birth, maternal bed sharing with the newborn, feeding on demand), support some recommendations in the ‘Ten Steps to Successful Breastfeeding’, including ‘support mothers to initiate breastfeeding as soon as possible after birth’, ‘enable mothers and their infants to remain together and to practise rooming-in 24 hours a day’, and ‘support mothers to recognize and respond to their infants’ cues for feeding’ [6].

### Breastfeeding practices of Chinese mothers from mainland China versus mothers from Hong Kong

Breastfeeding practices vary geographically in China [15]. This study reveals lower rate of breastfeeding initiation and shorter breastfeeding duration among mothers born in Hong Kong, in comparison to mothers born in Mainland China. Our results are consistent with the literature that the ‘ever breastfed’ rate in Hong Kong (< 67%, from population-based cross-sectional or cohort studies [72–76]) is lower than that in Mainland China (> 80% overall, from a review article [15]). Being separated from Mainland China for more than one century (1841–1997), the Hong Kong society has different health beliefs and lifestyle in comparison to the Mainland Chinese. Breastfeeding were found to be tied to social class in Hong Kong. Working women were given much more status and mothering seen as secondary [21]. Tarrant et al. [77] indicated that breastfeeding was viewed as a private act best practised away from the public settings. Advertisement on infant formula was prevalent in the mass media while promotion of breastfeeding was rare [77]. Our results could also be explained by the previous analyses of the same sample that immigrant mothers born in Hong Kong have less knowledge, less positive attitudes and weaker cultural beliefs about breastfeeding than immigrant mothers born in Mainland China [23].

### Strengths

Most of the migration studies on the breastfeeding used a single research method. The mixed method design is a distinctive strength of this study as it provides triangulation of data. Moreover, a number of measures have been used to ensure the rigorousness of our study, including blind-back translation of the questionnaire in the quantitative phase, and tape-recording, verbatim transcription, field notes and respondent validation in the qualitative phase.

### Limitations

Limitations of this study should be acknowledged. First, owing to the nature of the study, social desirability bias might exist in both study phases. Second, no information on exclusive breastfeeding was documented. Third, recall bias might exist in the cross-sectional retrospective survey, due to the 6-year gap between childbirth/breastfeeding and our assessment. However, our quantitative study design is considered as the most suitable approach, because the birth rate of the Chinese immigrants in Ireland is low, making a birth cohort study impossible. Further, a recent validation study conducted in the US demonstrates that maternal recall of breastfeeding duration is valid 6 years after childbirth [78]. Earlier studies in Australia reveal that the duration of breastfeeding could be remembered accurately up to 10 years [79, 80]. Hence, our data on breastfeeding duration may be considered as relatively accurate as the index child is 5 to 6 years on average. Moreover, a number of variables (e.g., time to introduce first formula, time to initiate breastfeeding, where feeding information was obtained, maternal breastfeeding intention) are less reliable owing to recall bias, leading to bias in the determinants of breastfeeding initiation and duration. Finally, a major limitation of this paper is that the data was collected about 10 years ago. Breastfeeding practices of the Chinese in Ireland and their influential factors may have changed, because efforts have been put to promote breastfeeding worldwide [7]. Enhancing breastfeeding support skills of the healthcare professionals, developing lactation specialists, and strengthening antenatal education have been taken into action in Ireland [7]. Except from the current study, no information on breastfeeding practices of the Chinese in Ireland has been reported. According to the Perinatal Statistics Report 2014 (53% of the Asian immigrants in Ireland breastfeed exclusively) [25], we speculate some improvement in breastfeeding practices among the Chinese in Ireland in the recent 10 years. As indicated previously, breastfeeding rates of non-nationals are higher than that of the Irish nationals [25, 32]. There are no specific strategies or actions to support non-nationals in Ireland. Barriers specific to non-nationals have not been addressed, such as the need for culturally sensitive and linguistic support of breastfeeding [7]. As a result, we believe that the factors influencing breastfeeding highlighted in this study should still be emphasized.

## Conclusions

This is the first and only migration study on breastfeeding among the Chinese in Ireland, reporting that giving birth in Ireland was associated with a shorter duration of breastfeeding. Reasons for discontinuing breastfeeding revealed here (including cultural conflicts, a lack of family support, linguistic isolations, and mothers’ low socio-economic status in Ireland) suggest a need for culturally and linguistically sensitive breastfeeding support in Ireland. The mixed methods design presented in this paper might serves as a template for future migration research on breastfeeding.

## Data Availability

The datasets used and/or analyzed during the current study are available from the corresponding author on reasonable request.

## Appendix

**Table 6:** Variables assessed in relation to breastfeeding in Phase 1 of this study^a^

	Total population	CMC	CMI
	(*n* = 322)	(*n* = 47)	(*n* = 275)
	No. (%)	No. (%)	No. (%)
Biomedical factors			
Obstetric experience			
Spontaneous vaginal delivery (SVD)	260 (81.0)	32 (68.1)	228 (83.2)
Caesarean section	61 (19.0)	15 (31.9)	46 (16.8)
Gestational age			
< 37 weeks	23 (7.2)	3 (6.4)	20 (7.3)
37–42 weeks	264 (82.2)	37 (78.7)	227 (82.8)
> 42 weeks	34 (10.6)	7 (14.9)	27 (9.9)
Maternal health condition at time of childbirth^c^			
Unhealthy	18 (5.6)	1 (2.1)	17 (6.2)
Healthy	303 (94.4)	46 (97.9)	257 (93.8)
Child’s health condition at time of childbirth^c^			
Unhealthy	26 (8.1)	1 (2.1)	25 (9.1)
Healthy	295 (91.9)	46 (97.9)	249 (90.9)
Behavioural factors			
Mother’s previous breastfeeding experience			
Had given birth to and breastfed the other child (ren)	75 (23.4)	0 (0)	75 (27.85)
Had given birth to but not breastfed any other child (ren)	29 (9.1)	2 (4.3)	27 (9.9)
Did not have birth nor breastfeeding experience	216 (67.5)	45 (95.7)	171 (62.6)
Newborn bed shared with mother			
Yes	89 (27.7)	21 (44.7)	68 (24.8)
No	232 (72.3)	26 (55.3)	206 (75.2)
Type of milk feeding			
On demand	124 (38.6)	17 (36.2)	107 (39.1)
In routine milk	197 (61.4)	30 (63.8)	167 (60.9)
1st time to breastfeed			
≤ 1 h after childbirth	103 (32.7)	8 (17.0)	95 (35.4)
> 1 h or never	212 (67.3)	39 (83.0)	173 (64.6)
1st time to feed with formula			
≥ 1 week or never	115 (36.1)	32 (68.1)	83 (30.5)
< 1 week	204 (63.9)	15 (31.9)	189 (69.5)
Timing of the introduction of other non-milk-liquids			
≥ 1 week or never	289 (93.8)	32 (71.1)	257 (97.7)
< 1 week	19 (6.2)	13 (28.9)	6 (2.3)
Maternal consumption of the cultural postpartum diet			
Yes	173 (53.7)	35 (74.5)	138 (50.2)
No	149 (46.3)	12 (25.5)	137 (49.8)
Social support and influence			
Feeding information obtained from internet			
Yes	79 (24.6)	7 (14.9)	72 (26.3)
No	242 (75.4)	40 (85.1)	202 (73.7)
Feeding information obtained from mother’s own mother			
Yes	178 (55.5)	20 (42.6)	158 (57.7)
No	143 (44.5)	27 (57.4)	116 (42.3)
Feeding information obtained from antenatal classes			
Yes	97 (30.2)	23 (48.9)	74 (27.0)
No	224 (69.8)	24 (51.1)	200 (73.0)
Her own mother’s attitude influenced her feeding choice			
Yes	114 (35.6)	14 (29.8)	100 (36.6)
No	206 (64.4)	33 (70.2)	173 (63.4)
Health professionals’ attitude influenced mother’s feeding choice			
Yes	79 (24.7)	12 (25.5)	67 (24.5)
No	241 (75.3)	35 (74.5)	206 (75.5)
Family type at time of childbirth			
Extended family	86 (26.9)	27 (57.4)	59 (21.6)
Nuclear family	234 (73.1)	20 (42.6)	214 (78.4)
Child was looked after by maternal grandmother			
Yes	39 (12.1)	9 (19.1)	30 (10.9)
No	283 (87.9)	38 (80.9)	245 (89.1)
Child was looked after by paternal grandmother			
Yes	36 (11.2)	14 (29.8)	22 (8.0)
No	286 (88.8)	33 (70.2)	253 (92.0)
Timing of return to work after childbirth			
Before 4 months	55 (17.2)	13 (27.7)	42 (15.4)
4–6 months	86 (26.9)	14 (29.8)	72 (26.4)
7–12 months	53 (16.6)	7 (14.9)	46 (16.8)
> 12 months or went back to work	126 (39.4)	13 (27.7)	113 (41.4)
Attitudinal factors			
Feeding decision was made			
Before pregnancy	155 (49.1)	18 (39.1)	137 (50.7)
During pregnancy	109 (34.5)	18 (39.1)	91 (33.7)
After the child was born	52 (16.5)	10 (21.7)	42 (15.6)
Planned breastfeeding duration			
≥ 4 months	189 (58.7)	38 (80.9)	151 (54.9)
< 4 months/never	133 (41.3)	9 (19.1)	124 (45.1)
Attitude towards colostrum			
Important for the baby	276 (86.2)	42 (89.4)	234 (85.7)
Harmful for the baby/Should be discard/Don’t know	44 (13.8)	5 (10.6)	39 (14.3)
I do not like breastfeeding			
Disagree	248 (78.7)	38 (80.9)	210 (78.4)
Agree/Don’t know	67 (21.2)	9 (19.1)	58 (21.7)
Formula feeding is more convenient than breastfeeding			
Disagree	133 (42.0)	26 (55.3)	107 (39.6)
Agree/Don’t know	184 (58.0)	21 (44.7)	162 (60.4)
I would feel embarrassed if someone saw me breastfeeding			
Disagree	146 (46.1)	27 (57.4)	119 (44.1)
Agree/Don’t know	171 (53.9)	20 (42.6)	151 (55.9)
Some traditional Chinese food can help to improve milk production			
Agree	271 (85.2)	41 (89.1)	230 (84.6)
Disagree/Don’t know	47 (14.8)	5 (10.9)	42 (15.4)
	Mean ± S.D.	Mean ± S.D.	Mean ± S.D.
Maternal awareness (score) of the advantage of breastfeeding for the baby^c^	4.04 ± 0.73	4.22 ± 0.69	4.00 ± 0.74

*CMC* Chinese mother gave birth in China, CMI Chinese mother gave birth in Ireland

Columns where the numbers do not add up to the specific n reflect missing values for this column

^a^This table excluded socio-demographic variables which were listed in Table 1

^c^Maternal awareness of the advantages of breastfeeding was assessed by a scale created by the investigator of this study after a review of the relevant literature. Details have been described by Zhou et al. [23]. It was a 5-point Likert scale (Strongly disagree = 1; Disagree = 2; Neither = 3; Agree = 4; Strongly agree = 5) via four statements: S1. A breastfed baby is likely to have fewer infections than a formula fed baby; S2. Breast milk is the ideal food for babies; S3. Breastfeeding provides health benefits for infants that cannot be provided by formula milk; and S4. Breastfeeding significantly reduces the risk of a large number of infant diseases. Score of awareness was calculated by summing all awareness scale items and dividing by the total number of items. Cronbach’s alpha = 0.836, indicating acceptable reliability

^c^Unhealthy: diagnosed as having any illness; Healthy: diagnosed as not having any illness

**Table 7:** Significant association (*P* < 0.15) between factors and breastfeeding initiation among CMI, revealed by univariate logistic regression analyses (Results of Phase 1)

	Initiated breastfeeding	Unadjusted OR (95% CI)
	Mean ± SD	
Socio-demographic factors		
Length of Irish residency at time of interview (years)	7.8 ± 5.07	0.95 (0.902–0.996)*
Child’s current age (years)	3.8 ± 3.34	0.90 (0.84–0.97)*
	n (%)	
Mother‘s birthplace		
Mainland China	184 (78.3)	2.62 (1.28–5.36)*
Hong Kong	22 (57.9)	1
Education level		
Tertiary	117 (82.4)	2.16 (1.23–3.80)*
Primary/Secondary	91 (68.4)	1
Current occupation		
Self-employed/ Professional work	54 (87.1)	3.01 (1.30–6.95)*
Non-professional work	69 (76.7)	1.47 (0.79–2.73)
Housewife	83 (69.2)	1
Mother has been breastfed		
Yes	183 (78.9)	2.69 (1.36–5.32)*
No/Don’t know	25 (58.1)	1
Husband’s education level		
Tertiary	92 (82.1)	1.88 (1.04–3.39)*
Primary/Secondary	115 (71.0)	1
Parity		
Primiparous	137 (80.1)	1.87 (1.07–3.27)*
Multiparous	71 (68.3)	1
Current accommodation		
Rented	150 (79.4)	0.53 (0.30–0.94)*
Family’s own property	57 (67.1)	1
Husband’s current occupation		
Self-employed/ Professional work	72 (82.8)	0.55 (0.29–1.06)
Non-professional work/ Unemployed	133 (72.7)	1
Child’s gender		
Female	101 (79.5)	1.53 (0.87–2.69)
Male	104 (71.7)	1
Behavioural factors		
Maternal breastfeeding experience		
Had given birth to and breastfed the other child (ren)	61 (81.3)	1
Had given birth to but not breastfed any other child (ren)	10 (37.0)	0.14 (0.05–0.36)*
Did not have birth nor breastfeeding experience	137 (80.1)	0.93 (0.46–1.85)
Newborn bed shared with the mother		
Yes	56 (82.4)	1.70 (0.85–3.41)
No	151 (73.3)	1
Social support and influence		
Feeding information obtained from internet		
Yes	64 (88.9)	3.28 (1.48–7.26)*
No	144 (70.9)	1
Feeding information obtained from antenatal classes		
Yes	63 (85.1)	2.21 (1.09–4.50)*
No	145 (72.1)	1
Feeding information obtained from mothers’ own mother		
Yes	129 (81.6)	
No	78 (67.2)	1
Her own mother’s attitude influenced her feeding choice		
Yes	86 (84.3)	2.25 (1.20–4.20)*
No	122 (70.5)	1
Health professionals’ attitude influenced mother’s feeding choice		
Yes	63 (91.3)	4.42 (1.82–10.75)*
No	145 (70.4)	1
Attitudinal factors		
Feeding decision was made		
Before pregnancy	125 (88.0)	11.95 (5.35–26.67)*
During pregnancy	67 (73.6)	4.54 (2.08–9.88)*
After the child was born	16 (38.1)	1
Attitude towards colostrum		
Important for the baby	192 (82.1)	7.14 (3.51–14.54)*
Harmful for the baby/Should be discard/Don’t know	16 (39.0)	1
I do not like breastfeeding.		
Disagree	179 (85.2)	7.17 (3.86–13.33)*
Agree/Don’t know	29 (44.6)	1
Formula feeding is more convenient than breastfeeding.		
Disagree	91 (85.0)	2.48 (1.33–4.63)*
Agree/Don’t know	117 (69.6)	1
I would feel embarrassed if someone saw me breastfeeding.		
Disagree	98 (82.4)	1.96 (1.09–3.50)*
Agree/Don’t know	110 (70.5)	1
Some traditional Chinese food can help to improve milk production.		
Agree	181 (78.7)	2.46 (1.25–4.84)*
Disagree/Don’t know	27 (60.0)	1
	Mean ± SD	
Awareness of the advantages of breastfeeding (score)	4.1 ± 0.69	2.46 (1.64–3.67)*

**P* < 0.05

Biomedical factors were not included in this table as none of the variables had a significant association (P < 0.15) with breastfeeding initiation among CMI

**Table 8:** Significant association (*P* < 0.15) between factors and breastfeeding duration among CMI, revealed by univariate logistic regression analyses (Results of Phase 1)

	Breastfed ≥4 months	Unadjusted OR (95% CI)
	Mean ± SD	
Socio-demographic factors		
Mother’s age at time of childbirth (years)	28.9 ± 4.72	1.05 (0.99–1.11)
Length of Irish residency at time of interview (years)	7.4 ± 4.78	0.95 (0.897–1.001)
Child’s current age (years)	8.1 ± 5.08	0.91 (0.85–0.99)*
	n (%)	
Mother‘s birthplace		
Mainland China	98 (41.7)	2.31 (1.04–5.09)*
Hong Kong	9 (23.7)	1
Education level		
Tertiary	71 (50.0)	2.69 (1.63–4.46)*
Primary/Secondary	36 (27.1)	1
Current occupation		
Self-employed/ Professional work	30 (48.4)	1.95 (1.04–3.65)*
Non-professional work	38 (42.2)	1.52 (0.86–2.67)
Housewife	39 (32.5)	1
Mother has been breastfed		
Yes	100 (43.1)	3.90 (1.67–9.12)*
No/Don’t know	7 (16.3)	1
Husband’s education level		
Tertiary	56 (50.0)	2.18 (1.32–3.58)*
Primary/Secondary	51 (31.5)	1
Husband’s occupation		
Self-employed/ Professional work	42 (48.3)	1.74 (1.03–2.92)*
Non-professional work/ unemployed	64 (35.0)	1
Husband’s nationality		
Chinese	87 (36.2)	1
Irish	12 (54.5)	2.11 (0.88–5.09)
Other nationality	7 (63.6)	3.08 (0.88–10.81)
Social support and influence		
Feeding information obtained from mother’s own mother		
Yes	74 (46.5)	2.19 (1.32–3.65)*
No	33 (28.4)	1
Feeding information obtained from antenatal classes		
Yes	37 (50.0)	1.87 (1.09–3.21)*
No	70 (34.8)	1
Child was looked after by maternal grandmother		
Yes	17 (56.7)	2.52 (1.05–4.85)*
No	90 (36.7)	1
Child was looked after by paternal grandmother		
Yes	4 (18.2)	0.32 (0.11–0.98)*
No	103 (40.7)	1
Behavioural factors		
Maternal breastfeeding experience		
Had given birth to and breastfed the other child (ren)	33 (44.0)	1
Had given birth to but not breastfed any other child (ren)	3 (11.1)	0.16 (0.04–0.57)*
Did not have birth nor breastfeeding experience	71 (41.5)	0.90 (0.52–1.56)
Newborn bed shared with mother		
Yes	37 (54.4)	2.34 (1.34–4.08)*
No	70 (33.8)	1
1st time to breastfeed		
≤ 1 h after childbirth	56 (58.9)	3.63 (2.16–6.12)*
> 1 h or never	51 (28.3)	1
1st time to feed with formula		
≥ 1 week or never	53 (63.9)	4.52 (2.61–7.80)*
< 1 week	54 (28.1)	1
Child was fed		
On demand	59 (55.1)	3.07 (1.85–5.10)*
In routine milk	48 (28.6)	1
Cultural postpartum diet consumption		
Yes	75 (54.3)	3.91 (2.33–6.56)*
No	32 (23.4)	1
Attitudinal factors		
Feeding decision was made		
Before pregnancy	71 (50.0)	13.00 (3.84–44.02)*
During pregnancy	33 (36.3)	7.40 (2.12–25.81)*
After the child was born	3 (7.1)	1
Planned breastfeeding duration		
≥ 4 months	95 (62.9)	15.83 (8.02–31.28)*
< 4 months/never	12 (9.7)	1
I do not like breastfeeding.		
Disagree	99 (47.1)	6.36 (2.89–13.97)*
Agree/Don’t know	8 (12.3)	1
Formula feeding is more convenient than breastfeeding.		
Disagree	58 (54.2)	2.88 (1.73–4.77)*
Agree/Don’t know	49 (29.2)	1
I would feel embarrassed if someone saw me breastfeeding.		
Disagree	58 (48.7)	2.08 (1.27–3.40)*
Agree/Don’t know	49 (31.4)	1
	Mean ± SD	
Awareness of the advantages of breastfeeding (score)	4.2 ± 0.64	2.12 (1.46–3.08)*

**P* < 0.05

Biomedical factors were not included in this table as none of the variables had a significant association (*P* < 0.15) with breastfeeding duration among CMI

## Abbreviations

-2LL: Log-likelihood statistics
CI: Confidence Interval
CMC: Chinese mothers (living in Ireland) who gave birth to the index child in China
CMI: Chinese mothers (living in Ireland) who gave birth to the index child in Ireland
HSE: Health Service Executive
OR: Odds Ratio
P: Significance value
SD: Standard Deviation
SPSS: Statistical Package for Social Sciences
WHO: World Health Organization
